# Determination and Comparison of Affecting Two Methods of Self-aid and Body-aid Based on the Multimedia Software Packages Method and the Lecturing on the Amount of Knowledge and Operation of Personals of Selected Combat Battalions

**DOI:** 10.5812/ircmj.2461

**Published:** 2013-05-05

**Authors:** Mohammad Daneshmandi, Davood Tadrisi, Ali Asgari, Jamileh Mokhtari, Abbas Ebadi

**Affiliations:** 1 Faculty member, Faculty of Nursing, Baqiyatallah University of Medical Sciences, Tehran, IR Iran; 2 Faculty of Nursing, Baqiyatallah University of Medical Sciences, Tehran, IR Iran

**Keywords:** First Aid, Teaching

## Dear Editor,

The present research is a quasi-experimental study with two groups (before and after training). This study compared the two methods of lecturing training and education by the multimedia software. Two selected battalions were considered in which each battalion had 300 personals. Based on the entry and exit criteria and preliminary test of measuring the knowledge (cognitive domain) (1). Those personals were selected whose scores were less than 60% of the test (200 in the first group and 180 from the second group). 30 personals were selected randomly organized from each battalion. These personals were trained with self-aid and body-aid techniques by using the lecturing method and multimedia software training. The necessary sample size in each group was estimated by Altman Nomogram (2), about 28 cases respectively. 30 cases were calculated with the probability of 10% detachment in the sample size. Namely, a total of 60 personals were considered in both groups. The initial conditions for participation in the statistical community were the followings: diploma education and above, obtaining preliminary test scores of less than 60%, no previous training in self-aid and body-aid, to be whether in the first or second decades of service. These cases were also the initial conditions for participation in the statistical community of multimedia software packages group: minimal familiarity with Microsoft Windows software, having a personal computer and the ability to use a multimedia CD (3). The pre-test in both groups was consisted of 38 written standardized questions. The validity and reliability were assessed as formal and substantive. The questions related to the cognitive domains (knowledge) were performed before the intervention (4). Then, the face to face training was conducted for the first group (5). This training was included the followings: lecture, questioning and answering and self-aid and body-aid educational booklet (prepared and notified by Training Deputy Joint Staff of the IRGC). The non-attendance training was considered for the second group. The educational package was provided for this group containing the following items: multimedia educational CD with the content of self-aid and body-aid (prepared and notified by Training Deputy Joint Staff of the IRGC) (6). The reliability and validity was confirmed by the editorials. The content of this educational package included: literature, printed CD, slides, images, audio, video and tests. Teaching was performed without limits of time and the place (7). The time interval between the intervention and holding the pre-test in both groups was one week after the end of face to face teaching and distribution of training package for non-attendance teaching. The pre - test was included 38 written questions. The questions were used in the cognitive domain (knowledge) and 8-station objective structured clinical examination (to assess functional areas) in face to face teaching and non-attendance groups. Using independent t-test (8), the level of knowledge was compared in both groups before and after the intervention. The results of Table 1 show that the mean level of knowledge has been significantly increased after the training in both of face to face teaching and multimedia groups (P < 0.001). However, the difference in scores (increase) is in favor of a multimedia teaching. The above table shows that there is no meaningful difference in the stations of 1, 5, 7 and 8 in both groups. There is meaningful difference in the stations of 2, 3, 4 and 6 in both groups. The status of the lecturing group was better in station 2, but in contrast, the multimedia group status was better in stations 3, 4 and 6 (Table 1).

**Table 1.** Comparison of the Mean Level of Knowledge Before and After Training

Knowledge level Group	Before, Mean ± SD	After, Mean ± SD	Significant level (paired T test)
**Lecturing**	18.9 ± 3	23.7 ± 2.9	P < 0.001
**Multi media**	17.2 ± 3.9	28.2 ± 2.5	P < 0.001
**Significant level (independent t)**	0.07	P < 0.001	
**Group After Station**	Lecturing, Mean ± SD	Multimedia Mean ± SD	Significant level (Man- Whitney test)
**One**	90.0 ± 14.3	89.2 ± 11.1	> 0.05
**Two**	88.5 ± 13.8	71.4 ± 10.7	< 0.001
**Three**	51.7 ± 11.7	66.2 ± 6.9	< 0.001
**Four**	58.3 ± 16.5	80.8 ± 9.7	< 0.001
**Five**	71.2 ± 20.8	72.9 ± 13.9	> 0.05
**Six**	51.9 ± 15.6	80.0 ± 10.3	< 0.001
**Seven**	89.5 ± 10.9	82.5 ± 14.8	> 0.05
**Eight**	65.1 ± 7.9	60.0 ± 10.8	> 0.05

The results of this study have been confirmed the hypothesis (the superiority of multimedia software training than the lecturing teaching method and more influence on the rate of learning) of the study. This study showed that in the case of optimum conditions regarding the space and time, multimedia training software can be more effective than the face to face teaching methods. Therefore, it is suggested to use this method in order to save time and cost of the in-service training personals.
